# A novel scoring protocol reveals age-related differences in abstract compared to concrete thinking in cued autobiographical remembering

**DOI:** 10.1038/s41598-024-82493-6

**Published:** 2024-12-27

**Authors:** Mariam Hovhannisyan, Quentin Raffaelli, Nadine Chau, Jessica R. Andrews-Hanna, Matthew D. Grilli

**Affiliations:** 1 Department of Psychology, University of Arizona, 1503 E University Blvd, Tucson, AZ 85721 USA; 2 Cognitive Science, University of Arizona, Tucson, AZ USA; 3 Evelyn F. McKnight Brain Institute, University of Arizona, Tucson, AZ USA; 4 Department of Neurology, University of Arizona, Tucson, AZ USA

**Keywords:** Cognitive ageing, Psychology, Human behaviour

## Abstract

Human imagination has garnered growing interest in many fields. However, it remains unclear how to characterize different forms of imaginative thinking and how imagination differs between young and older adults. Here, we introduce a novel scoring protocol based on recent theoretical developments in the cognitive neuroscience of imagination to provide a broad tool with which to characterize imaginative thinking. The scoring protocol distinguishes between concrete/perceptual forms of imagination termed the “mind’s eye” and abstract/reflective forms of imagination termed the “mind’s mind.” The protocol also captures whether thoughts pertain to the self, others, or both. We applied this scoring protocol with high inter-rater reliability across two studies involving distinct participants and narrative-based imagination tasks. When compared to young adults, older adults showed a bias toward general content, which is a feature of the mind’s mind form of thinking while describing aloud their memories of specific, past events (Study 1). Further, older adults made fewer references to the self. In a separate study of only older adults (Study 2), increasing age was not associated with a bias toward the mind’s mind while describing specific past or future events. These results reveal that imaginative thinking can be characterized within the Mind’s Eye Mind’s Mind framework, with implications for understanding cognitively normal older age.

**Supplementary Information:**

The online version contains supplementary material available at 10.1038/s41598-024-82493-6.

## Introduction

Human imagination, the ability to form internal images or ideas not present to the senses^1^, allows us to mentally transcend space and time to remember the past, think about the future, and engage in many other forms of thinking^2^. While a large body of research has examined these different manifestations of imagination, we recently proposed a broader neurocognitive organization differentiating distinct component processes of imagination referred to as the “mind’s eye” (concrete, image-based) and the “mind’s mind” (abstract, verbal-based)^3,4^. In the present study, we use the Mind’s Eye Mind’s Mind framework, first, to develop a novel scoring protocol to quantify these processes and second, to gain insight into how past and future thinking may differ between young and older adults.

A common thread among theories of imaginative thinking is that this form of cognition is an outcome of multiple processes that can interact or operate independently^3–7^. Drawing on the episodic and semantic memory distinction^8^, researchers note, for example, that theorized processes underlying episodic memories, such as perceptual processing^5,6^ and relational binding^9^, may be important for past and future thinking. Researchers have made a similar claim for semantic memory, with conceptual, abstract, and reflective thinking identified as candidate processes of imagination^5,10–12^. Other views draw less from the episodic and semantic dichotomy, and instead offer that self-projection^13^ or mental scene construction^14,15^ are central to imagination. Observing the presumed neural correlates of these theorized processes, researchers have further proposed that the brain’s default network underlies many processes central to human imagination^4,16,17^.

Recently, we proposed that many of the ideas related to imagination and the default network appear to coalesce around two broad component processes that form the basis of human imagination^3^. In this overarching framework, the mind’s eye reflects imagination experienced at low levels of construal, including concrete and contextually specific thoughts, often using an image-based representational form. Neuroscience research suggests that the medial temporal lobe (MTL) subsystem of the default network^18^, also referred to as the posterior-medial episodic network^19^ and a closely aligned “DMN-A subsystem”^20^, plays an important role in mind’s eye forms of imaginative thinking^3–5^. In contrast, the mind’s mind is proposed to capture imaginative thought experienced at higher levels of construal, involving abstract and reflective forms of imagination more accessible to a verbal representational form. According to this framework, the dorsal medial prefrontal cortex (dmPFC) subsystem of the default network^18^, which is closely aligned with the "DMN-B subsystem"^20^, is principally linked to the mind’s mind component of imaginative thinking^3,4^.

Based on the Mind’s Eye Mind’s Mind framework, we can expect imagination to take one form or the other to varying degrees as a function of the involvement of the underlying subsystems. Neuropsychological cases have served as one source of supporting evidence for this claim. For instance, individuals with medial temporal lobe amnesia display impairments when past, future, and atemporal imagined thoughts involve constructing specific details^21–23^, visualizing scenes^24^, and retrieving visually complex or spatiotemporally specific knowledge^25–30^. Likewise, individuals with semantic dementia, which disproportionately affects the dmPFC subsystem, can comprehend concrete words better than abstract words^31^ and show less impairment in episodic memory compared to semantic memory^32^. Importantly, although neuropsychological cases can provide insight into extreme shifts in imagination, the Mind’s Eye Mind’s Mind framework proposes much broader state, trait, and life course variations in default network involvement that shape the form of imagined thought. One such life course stage, and the focus of the present study, is typical older age.

Research in psychology and neuroscience has raised the possibility that typical older age may show a natural, possibly adaptive, shift toward the mind’s mind in service of certain forms of imagination. Functional neuroimaging studies, for example, have shown that older adults, relative to young adults, tend to overactivate regions of the dmFPC subsystem, and underactivate regions of the MTL subsystem, while mentally constructing and elaborating past and future events^33,34^. Likewise, it is well established that older adults, when compared to young adults, provide less episodic or event-specific details and more semantic details while orally describing past and future events in laboratory settings^35,36^. In fact, these findings are commonly interpreted as evidence that older adults may be taking a more conceptual or reflective strategy toward event memory retrieval and future event thinking, perhaps due to a lack of access to specific information and/or a change in motivation that shifts imagination toward a high level of construal^37–39^.

Despite suggestive functional neuroimaging and cognitive aging findings, whether cognitively healthy older age is associated with a mind’s mind shift remains unclear. A major reason underlying this gap is that the field lacks a scoring protocol to assess the interactions between concrete and abstract information across the full spectrum of imaginative thought (e.g., event remembering, mind-wandering, self-reflection, among others). As such, the explanatory value of the Mind’s Eye Mind’s Mind framework for understanding typical older age, or other state, trait, and life course factors, remains unanswered. In the present study, we set out to begin closing this gap by creating a scoring protocol that can be applied to characterize the relative use of the mind’s eye and the mind’s mind.

The novel scoring protocol also captures a potential preference to relate imagined thoughts to either the self or others, which is relevant for another age-related shift that is understudied in the literature. In particular, older adults might show a bias toward thinking more about others (as opposed to the self), due to socioemotional goals that place importance on social relationships^40,41^. For example, recent work has shown that during less cognitively demanding tasks (e.g., mind wandering), the content of older adults’ thoughts are less self-related and more other-related compared to young adults^42,43^. Although research on the content of imaginative thoughts in older adults is limited, possible factors such as motivation/interest^44^, emotional-wellbeing^45^, and a positivity bias^46^ might contribute to differences between young and older adults in the content of imaginative thought^47^. In recognition of these findings on self-referential thought and socioemotional goals in aging, we may expect age-related differences in references to the self and to others in many forms of imaginative thought. Therefore, we incorporated self-other orientation in the novel scoring protocol.

Based on prior work examining various cognitive and neural aspects of imaginative thinking, the present set of studies tested four hypotheses about aging and imagination. First, compared to young adults, we hypothesized that cognitively healthy older adults would show a bias for the mind’s mind while engaging in imagination, as shown by how past events are remembered (Study 1). Second, we hypothesized that the shift toward the mind’s mind might continue to evolve in older adulthood, as shown by a cross-sectional relationship with age among a separate cohort of cognitively healthy older adults tasked with remembering past events and thinking about future events (Study 2). Third, we hypothesized that the propensity to use mind’s eye and mind’s mind would be consistent across past and future forms of imagination, given prior work showing consistency in episodic and semantic information in these two forms of thinking^48,49^, and our expectation that use of the mind’s eye and mind’s mind partly reflect trait-like qualities^3^. Finally, based on findings showing age-related differences in self and other focus in task-unrelated thought^42^, we hypothesized that older adults’ thoughts would shift to be focused more on others and less on the self when compared to young adults (Study 1), and in association with advanced age among older adults (Study 2).

### Study 1

### Methods

### Participants

The first study is a secondary analysis of a dataset composed of 103 participants, including 54 young adults (mean age = 22.5 years, mean edu = 15.2 years, number of males = 12) and 49 older adults (mean age = 69.5 years, mean edu = 17.5 years, number of males = 13) who were asked to retrieve autobiographical memories^50^. Demographic and neuropsychological data are shown in Table 1. Eligibility was determined based on an actuarial approach shown to improve cognitive screening of abnormal age-related cognitive decline^51^. In brief, individuals were considered cognitively impaired, and thus ineligible for the study, if either of the following were met: (1) they performed more than 1 standard deviation below the age-corrected (and education-corrected, if available) normative mean on two critical scores in one cognitive domain, or (2) they performed more than one standard deviation below the age-corrected (and education-corrected, if available) normative mean on three critical scores across three cognitive domains. None of the older adults in the current study met these criteria. All participants were screened for self-reported neurological disorders and history of psychiatric conditions that could have cognitive effects. Informed consent was obtained from all participants. All study procedures were performed in accordance with relevant guidelines and regulations and approved by the University of Arizona Institutional Review Board.

**Table 1:** Demographic data for both studies.

Demographics	Study 1		Study 2
Mean (SD)	Older (*n* = 49)	Young (*n* = 54)	Older (*n* = 82)
Age (yrs)	69.5 (5.9)	22.5 (4.2)	68.85 (5.78)
Sex (% female)	73%	78%	66%
Education (yrs)	17.5 (1.8)	15.2 (2.3)	16.82 (1.81)
WAIS-IV VCI	120.79 (10.68)	121.45 (14.07)	122.71 (10.84)
*Race*			
Black or African American	2	1	2
White	47	31	79
Asian	0	7	1
Native American	0	1	0
Other	0	14	0
*Ethnicity*			
Hispanic or Latino	0	13	3
Not Hispanic or Latino	49	41	79

### Procedures

### Autobiographical Interview task

Participants underwent procedures for the Autobiographical Interview^35^ online. Due to the COVID-19 pandemic, all participants were seen via Zoom Health (see Hernandez et al.^50^ , for validity and applicability of the virtual Autobiographical Interview, as well as results with the Autobiographical Interview scoring). There were no differences in the procedure of the Autobiographical Interview in person versus via Zoom, except that participants were at home during the interview. Each participant recalled five unique life events from multiple time periods (early childhood, adolescence, early adulthood, middle adulthood, and within the last year). Young adults were asked to recall two early adulthood memories rather than one from middle adulthood. Participants were instructed to focus on a specific event that occurred within 24 h (e.g., a vacation to Hawaii would not be adequate, but one day of that vacation would be). If a participant did not comprehend the instructions or provided a short description of the event, the experimenter gave a general probe (e.g., “Can you recall any other details?”). If a participant did not focus on a specific event, they were asked to select one to focus on (e.g., “Please choose one specific event to focus on”). Oral responses were recorded then transcribed. Participants had up to four minutes to provide their response, after which they were allowed to complete their thought and then signaled to stop if they were still talking. Raters did not score past the four-minute mark.

### Mind’s eye mind’s mind scoring protocol development

**Fig. 1:**
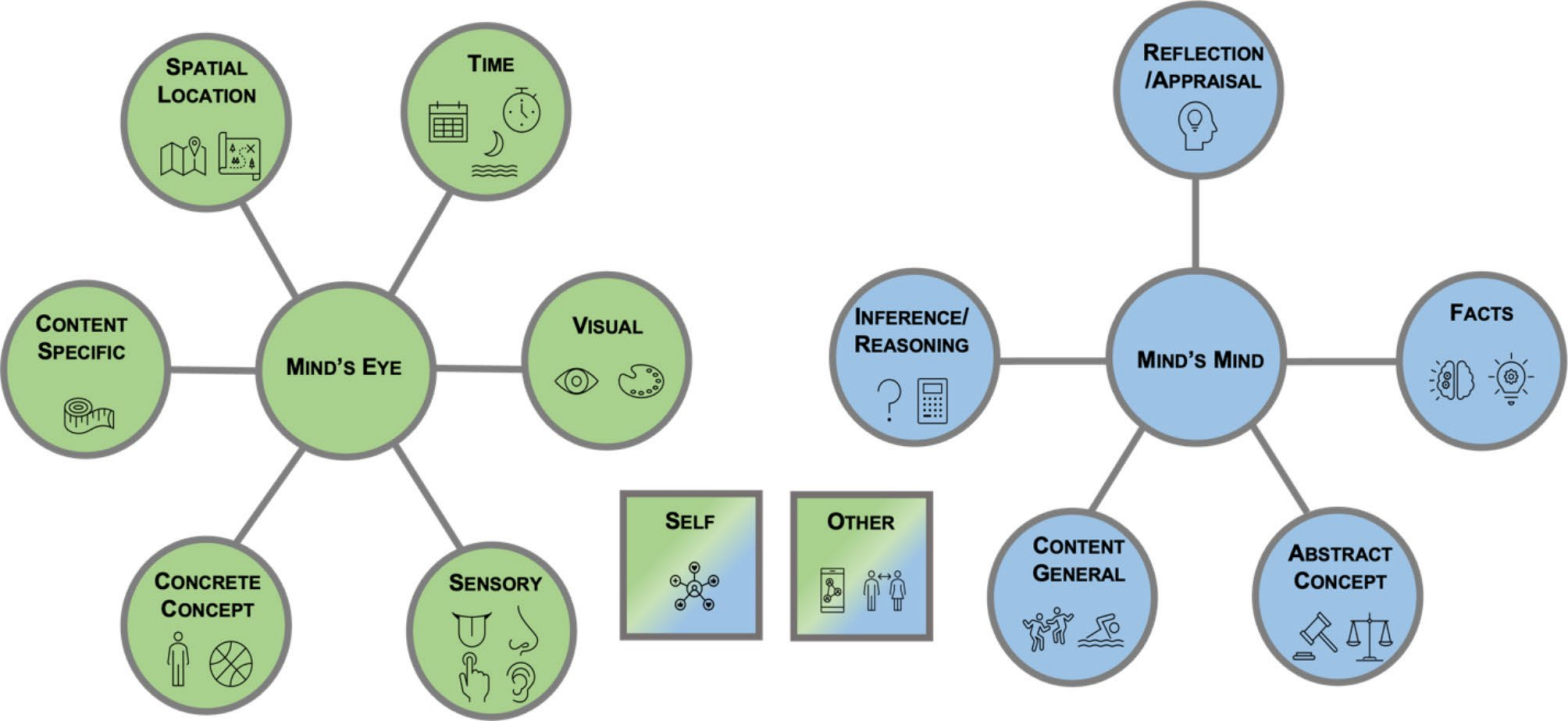
A matrix bubble showing mind’s eye and mind’s mind subtypes. Clusters categorized as mind’s eye (green) and mind’s mind (blue). Self- and other-related elements of thought can be used with mind’s eye or mind’s mind subtypes (blue/green gradient), but not on their own.

We developed the scoring protocol by defining imaginative subtypes that capture the essence of mind’s eye versus mind’s mind forms of imaginative thought. Although we designed the protocol to capture both prompted and unprompted imaginative thoughts, we applied the protocol to autobiographical past and future thoughts in the present studies. Based on prior theoretical and empirical research, we identified six imaginative subtypes (termed *elements of thought*) as forms of the mind’s eye category and five as forms of the mind’s mind category (see Table 2). The elements of thought within the mind’s mind category included content general elements, abstract concept elements, reflection/appraisal elements, inference/reasoning elements, and facts elements. The mind’s eye category consisted of content specific elements, concrete concept elements, spatial location elements, temporal elements, visual elements, and sensory elements. A visualization of the subtypes can be seen in Fig. 1 and descriptions of these subtypes and examples of each can be seen in Table 2. In addition, the scoring protocol also captures whether an element of thought (irrespective of its mind’s eye versus mind’s mind classification) was related to the self, others, or both. Our definition posits that the mind’s eye could feasibly be represented using an image-based representational form, whereas the general/abstract nature of the mind’s mind would be more accessible to a verbal representation. However, we do not exclude the possibility that these two forms of imagination interact. Our scoring protocol is developed with this idea in mind, meaning that the typical stream of thought manifests as an interactive mix of the mind’s eye and mind’s mind that unfold over time. For example, thinking about one’s dog might accompany an image of one’s own dog but also a reflective thought such as the feeling of love for one’s dog.

**Table 2:** Subtypes for both mind’s eye and mind’s mind, as well as self- and other- related elements. Labels used for scoring are provided in parenthesis.

	Short Definition	Example
**Mind’s Mind**		
Content General (CG)	Description of the event happenings, these are typically action statements that can include verbs	“We drove/CG home”
Abstract Concept (AC)	Non-tangible concepts, can also include figurative language	“We wanted justice/AC”
Reflection/Appraisal (REF-A)	Cognitive or emotional appraisals. Encompasses emotions, thoughts, opinions, and reflections	“He is kind/REF-A + O”
Inference/Reasoning (INF-R)	An inference that is made through a logical series of events, deductive or inductive reasoning, theory of mind, and guessing	“It is probably/INF-R going to rain”
Facts (F)	General, culturally shared knowledge	“The US flag has/F 50 stars”
**Mind’s Eye**		
Content Specific (CS)	An embellishment of something, a specifier	“We went to a foreign/CS market”
Concrete Concept (CC)	A tangible thing, including people	“The ball/CC went over the fence”
Spatial Location (LOC)	Any orientation to, or indication of, a location, space, or place	“I’m going to Tucson/LOC”
Time (T)	Any orientation to, or indication of, time, time passing, dates, and age	“In the morning/T”
Visual (V)	Description of visual features	“The glass/V door”
Sensory (SENS)	Sensory features including taste, touch, smell, hearing, hunger, pain, and temperature	“The stove was hot/SENS”
**Self-Other Orientation**		
Other (+ O)	Reference to others	“Julia/CC + O is a boss”
Self (+ S)	Reference to the self	“I/CC + S went home”

### Scoring procedures & psychometric properties

**Fig. 2:**
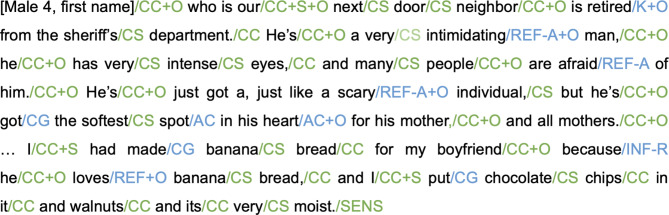
Partial transcript with novel scoring procedure applied. Green labels are mind’s eye elements and blue labels are mind’s mind elements.

A comprehensive manual of the mind’s eye and mind’s mind scoring protocol with specific examples of how it is applied to different types of thoughts can be found on OSF (https://osf.io/356kh/). We outline it here in three steps. First, the rater independently reads the entire narrative before scoring to get a sense of what the narrative is about and to understand the context. Second, the rater parses each narrative into elements of thought using slashes. An element of thought is often a grammatical clause, but it can also be a word. Lastly, the rater adds the element labels for mind’s eye and mind’s mind, including whether the element was related to the self (labelled “self”) and/or related to another person/other people (labelled “other”). Every thought element has only one corresponding label for mind’s eye and mind’s mind, and additional labels for self and/or other, if relevant (see Table 2). The self and other labels are not stand alone and are only added to other mind’s eye or mind’s mind elements. For example, “I went to the movies on Saturday” has three mind’s eye elements, one mind’s mind element, and one self/other element added to a mind’s eye element. Based on this scoring protocol: “I” is a concrete concept (CC; mind’s eye) related to the self (+ S), “went to” is content general (CG; mind’s mind) because it indicates a non-specific action, “the movies” is coded as a place (LOC; mind’s eye), and “on Saturday” is coded as time (T; mind’s eye). Non-imaginative thoughts and task-related inferences are defined as statements that reflected the present moment and included thoughts about the physical environment (e.g., “My dog just came into the room”), interoceptive perceptions (e.g., “I’m cold”), and thoughts about the task (e.g., “Am I finished?”). These were scored, but not considered part of mind’s eye or mind’s mind. See Fig. 2 for a longer example of how the scoring protocol is applied to the data.

Two trained raters (MLB and NC), who scored all the transcripts, worked independent of each other and were unaware of participant age. The interrater reliability between both raters, assessed by intraclass correlation coefficient (ICC)^52^ and interpreted based on Koo & Li’s^52^ guidelines, were excellent for both mind’s eye (ICC = 0.96) and mind’s mind (ICC = 0.93). The reliability was excellent for other-related elements (ICC = 0.98) and good for self-related elements (ICC = 0.86). The reliability for the non-imaginative thought category was poor (ICC = 0.45), likely due to these thoughts occurring less frequently. To create a more reliable estimate of the mind’s eye and mind’s mind thought elements, the two raters’ scores were averaged for each participant.

**Fig. 3:**
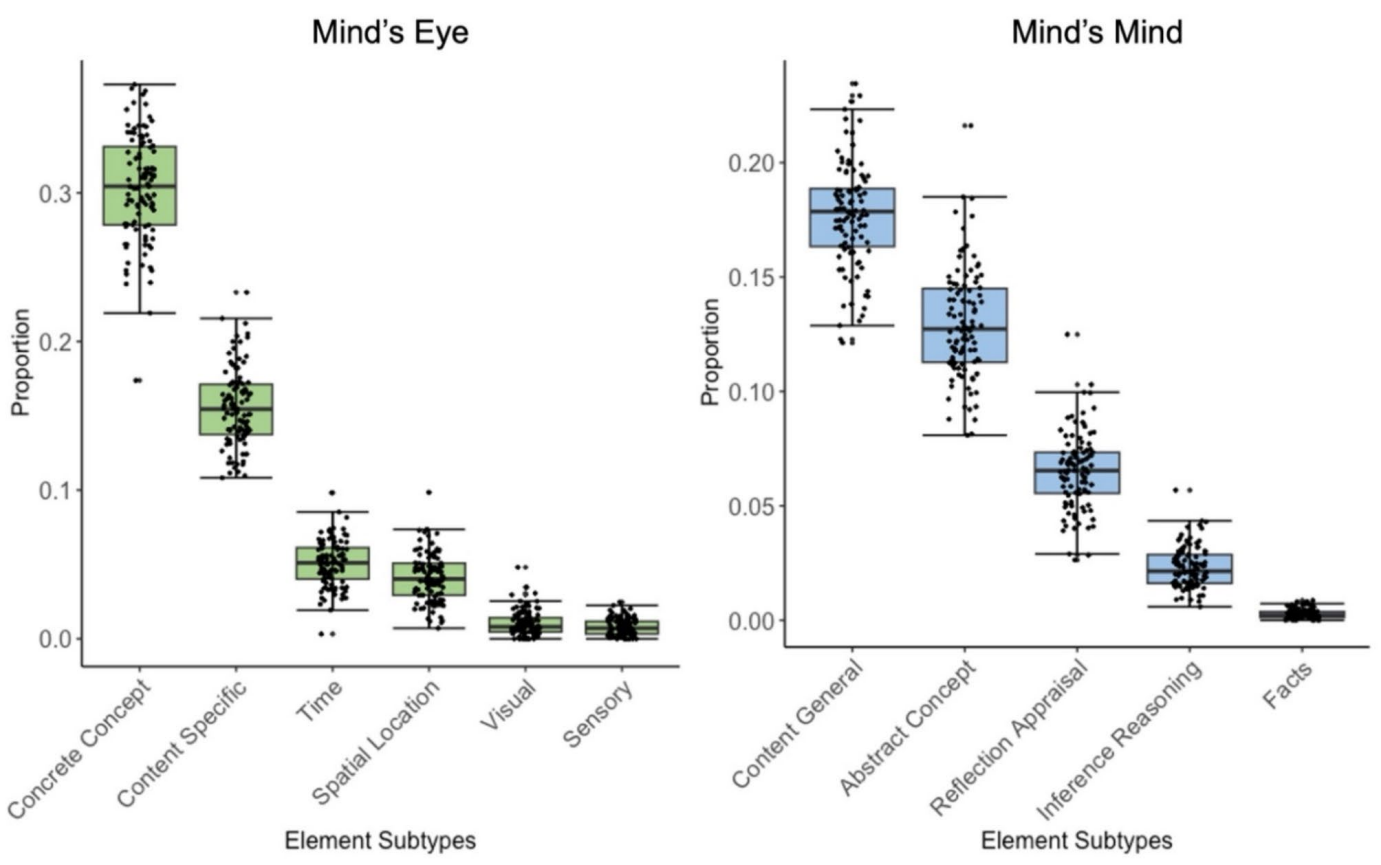
Frequency of the subtypes for Mind’s Eye and Mind’s Mind in Study 1. Boxplots show median represented by a line within each box. Brackets capture data within the upper and lower quartiles.

In addition to calculating reliability at the level of the mind’s eye and mind’s mind, we also calculate reliability for individual element subtypes. Table 3 shows the mean and standard deviation values for each subtype for each age group, as well as the intraclass correlation coefficient for each subtype. It is important to note that the facts subtype occurred at a much lower frequency than other subtypes in the present study, and thus has a lower intraclass correlation coefficient. All other element subtypes for mind’s eye and mind’s mind fall within a good to excellent classification^52^. Figure 3 demonstrates the most to least frequently occurring element subtypes for both mind’s eye and mind’s mind.

**Table 3:** Intraclass correlation coefficient and mean proportion values by subtype split by age group. ICC = intraclass correlation coefficient, M = mean, SD = standard deviation.

	ICC	OA		YA	
***Mind’s Mind***		M	SD	M	SD
Inference Reasoning	0.91	0.02	0.009	0.03	0.008
Abstract Concept	0.90	0.13	0.02	0.13	0.02
Content General	0.88	0.19	0.02	0.17	0.02
Reflection Appraisals	0.73	0.06	0.02	0.07	0.02
Facts	0.26	0.004	0.002	0.002	0.002
***Mind’s Eye***					
Content Specific	0.94	0.15	0.02	0.16	0.03
Concrete Concept	0.93	0.31	0.03	0.3	0.04
Visual	0.93	0.008	0.007	0.01	0.009
Location	0.92	0.04	0.02	0.04	0.02
Time	0.86	0.05	0.01	0.05	0.02
Sensory	0.73	0.008	0.005	0.008	0.006
***Other***					
Non-Imaginative	0.43	0.03	0.01	0.03	0.02

To further examine the psychometric properties of the scoring protocol we generated a correlation matrix using count data. The correlation matrix illustrates generally strong positive correlations across all element types, suggesting that many of the elements between and across the mind’s eye and mind’s mind are interactive (see Supplementary Figure S1). To provide additional clarity on the correlation matrix, we conducted an exploratory factor analysis (EFA) to assess the underlying structure of the data. Notably, the current study was poorly powered for an EFA of 11 element types, and therefore we interpret the results cautiously. The EFA was conducted with a varimax rotation on box-cox transformed count data. Parallel analysis^53^ was used to determine the number of factors, which revealed a two-factor solution accounting for 61% of the overall variance. Factor loadings can be seen in Supplementary Table S1. The factor loadings did not show a clear categorical separation of the mind’s eye and mind’s mind (see Discussion).

The current dataset was also scored with the Autobiographical Interview, which is commonly used to assess the specificity of event memories. Though there are similarities between the Autobiographical Interview scoring protocol and the Mind’s Eye Mind’s Mind scoring protocol, there are a few important differences. First, the element types vary between the scoring protocols and are applied differently to the data such that the current scoring protocol is applied within smaller units (see Fig. 2). Second, although we apply the Mind’s Eye Mind’s Mind scoring protocol to autobiographical memories and future events in this manuscript, the scoring protocol can be applied to other forms of imaginative thinking beyond specific events, such as resting state thoughts.

### Statistical analyses

Normality of the data was determined using skew and kurtosis values (with a cutoff of -1 to 1 for skew and − 2 to 2 for kurtosis). Effect sizes are reported as Cohen’s d for t-tests and generalized eta squared for analysis of variance (ANOVA) results. For ANOVAs, we used a Bonferroni correction on planned post hoc comparisons. All analyses were performed in R^54^. Figures were created in R using ggplot2^55^.

Study 1 tested two of our four hypotheses, namely that older adults, compared to young adults, have a bias to use the mind’s mind and think less about the self and more about others. We used two different measures to assess our hypotheses, a proportion measure (mind’s mind: total thought elements and other: total thought elements) and a ratio measure (mind’s mind: mind’s eye and other: self ). The proportion measure represents the count of thought elements within each category (mind’s eye and mind’s mind) out of the total number of thought elements (mind’s eye, mind’s mind, and non-imaginative/task-related inferences). The proportion, which provides a percentage measure accounting for non-imaginative thoughts, was highly correlated with the ratio measure (*r* (101) = 0.96 for mind’s eye and *r* (101) = 0.98 for mind’s mind, *p* values < 0.001), which compares the total mind’s mind elements to total mind’s eye elements and is another proxy for bias. For conciseness, we provide details of the ratio results in the Supplementary Information.

First, to examine the relative use of the mind’s eye and mind’s mind in young and older adults, we conducted a two-by-two mixed ANOVA using proportion values, with factors for category of imaginative thought (mind’s eye vs. mind’s mind) and age group (older vs. young adults). Second, to measure age group differences in the use of self-related versus other-related thoughts between young and older adults, we conducted a two-by-two mixed ANOVA using proportion values with factors for age group (older vs. young adult) and self-other orientation (self vs. other). We examined the correlation between the proportion and ratio measures and found that they were highly correlated for both self (*r* (101) = 0.80, *p* < .001) and other (*r* (101) = 0.91, *p* < .001). We provide the results of the ratio measure in the Supplementary Information.

In addition to these analyses, we also computed Pearson correlations between proportion scores for mind’s eye and mind’s mind, and internal and external details computed from the Autobiographical Interview. This allowed us to examine the shared variance between the Mind’s Eye Mind’s Mind scoring protocol and the Autobiographical Interview scoring protocol. As noted, there are both similarities and differences in the manner of organization of elements and details, and in the application of the scoring protocols (i.e., imaginative thinking broadly defined versus event memories/future thoughts). Given these similarities and differences, and in light of internal details and mind’s eye elements both capturing specific information, we hypothesized a small-to-moderate positive relationship between internal details and mind’s eye elements. We also predicted a small-to-moderate positive relationship between external details and mind’s mind elements given that they are both capturing semantic information, although the mind’s mind also captures other information such as making inferences or appraisals.

## Study 1 results

### Older adults show more of a tendency to use the mind’s mind compared to young adults

**Fig. 4:**
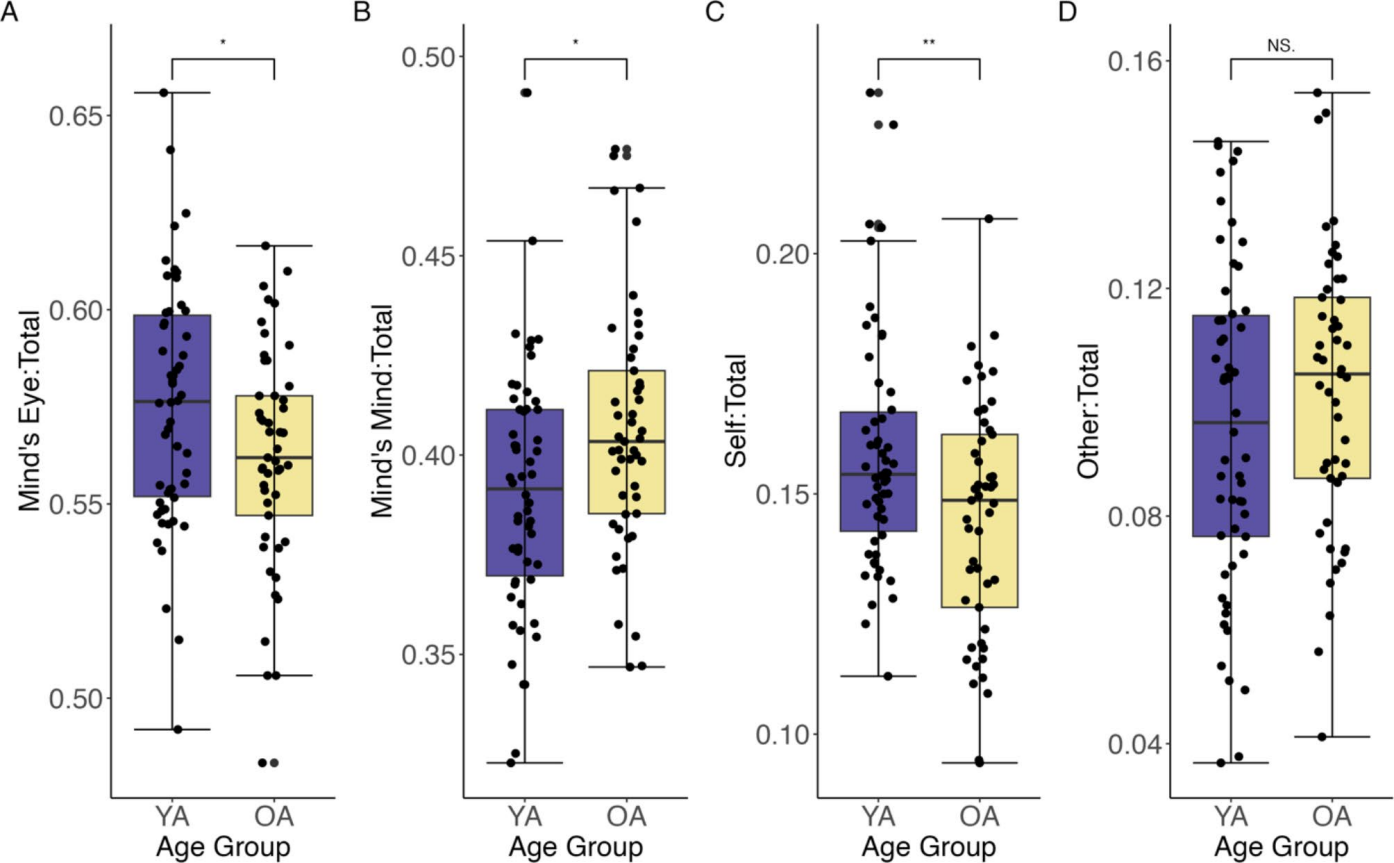
Study 1 age group differences for proportion of mind’s mind elements as well as self- and other-related elements. Age group differences for proportion of (**A**) mind’s eye elements, (**B**) mind’s mind elements, (**C**) self-related elements, and (**D**) other-related elements. Boxplots show median represented by a line within each box. Brackets capture data within the upper and lower quartiles.

Descriptive statistics for the proportion of mind’s mind and mind’s eye elements and ratio of mind’s mind to mind’s eye elements are reported in Supplementary Tables S2 and S3. Our two-way mixed ANOVA revealed a significant main effect for category of imaginative thought, *F* (1,101) = 843.19, *p* < .001, η^2^_*G*_ = 0.89, but not a significant effect of age group, *F* (1,101) = 0.64, *p* = .42, η^2^_*G*_ = 0.00034. Collapsing across age groups, proportion of mind’s eye elements (M = 0.57, SD = 0.03) was significantly greater than the proportion of mind’s mind elements (M = 0.40, SD = 0.03), *t* (102) = 28.5, *p* < .001, *d* = 2.81. There was also a significant category of imaginative thought by age group interaction, *F* (1,101) = 5.98, *p* = .016, η^2^_*G*_ = 0.053. Planned comparisons revealed that older adults exhibited a greater proportion of mind’s mind elements (M = 0.41, SD = 0.03) compared to young adults (M = 0.39 SD = 0.03), *t* (101) = 2.53, *p* = .013, *d* = 0.50, whereas young adults exhibited a greater proportion of mind’s eye elements (M = 0.58, SD = 0.03) compared to older adults (M = 0.56, SD = 0.03), *t* (101) = 2.22, *p* = .028, *d* = 0.44 (Fig. 4A and B). Results for the ratio measure for the mind’s mind to mind’s eye elements are consistent with the proportion findings and can be found in Supplementary Figure S2.

To examine whether the observed interaction between age group and imagination type was consistent across imagination thought subtypes, we conducted two separate mixed ANOVAs for mind’s mind and mind’s eye with factors for element subtypes and age group. A breakdown of proportion of subtypes by age group can be found in Table 3. The ANOVA for mind’s mind showed a significant main effect of age group, *F* (1,101) = 6.40, *p* = .013, η^2^_*G*_ = 0.009, a significant main effect of element subtype, F (4,404) = 1800.47, *p* < .001, η^2^_*G*_ = 0.94, and an interaction between age group and element subtype, *F* (4,404) = 5.72, *p* < .001, η^2^_*G*_ = 0.05. Planned comparisons using a Bonferroni correction revealed that older adults exhibited a greater number of content general elements compared to young adults, *t* (101) = 3.63, *p* < .001. There were no significant differences between young and older adults for all other element subtypes. To further analyze which element subtype occurs more frequently, we examined the main effect of subtype which showed that all comparisons between subtypes were significant using a Bonferroni correction for multiple comparisons, all *t*’s ≥ 14.4, *p’s* < 0.001. Content general elements occurred most frequently, followed by abstract concept, reflection/appraisal, inference/reasoning, and the least frequently occurring was facts (see Fig. 3).

The ANOVA for mind’s eye showed a significant main effect of age group, *F* (1,101) = 4.94, *p* = .03, η^2^_*G*_ = 0.003, and a significant main effect of element subtype, *F* (5,505) = 2825.72, *p* < .001, η^2^_*G*_ = 0.96, but there was not a significant interaction between age group and element subtype, *F* (5,505) = 1.03, *p* = .40, η^2^_*G*_ = 0.009. To examine which mind’s eye element subtype occurs more frequently, we examined the main effect of subtype which revealed that all but one comparison between subtypes were significant using a Bonferroni correction for multiple comparisons, all *t*’s ≥ 2.55, *p’s* < 0.001. The most frequently occurring element subtype was concrete concept, followed by content specific, time, spatial location, visual, and the least frequently occurring was sensory (see Fig. 3). The only comparison that was not significantly different was visual elements and sensory elements, *t* (103) = 2.6, *p* = .17.

### Older adults show less self-related thoughts compared to young adults

Descriptive statistics for the ratio of other- to self-related elements and proportion of self- and other-related elements and are reported in Supplementary Table S3 and S4. Regarding the use of the self and other in participants’ narrated autobiographical memories, the ANOVA revealed a significant main effect of self-other orientation, *F* (1,101) = 138.00, *p* < .001, η^2^_*G*_ = 0.51, but not a significant effect of age group, *F* (1,101) = 2.37, *p* = .13, η^2^_*G*_ = 0.005. Collapsing across age groups, proportion of self-related elements (M = 0.15, SD = 0.03) was significantly greater than the proportion of other-related elements (M = 0.10, SD = 0.03), *t* (102) = 11.65, *p* < .001. There was also a significant self-other orientation by age group interaction, *F* (1,101) = 4.96, *p* = .028, η^2^_*G*_ = 0.04. Planned comparisons revealed that older adults exhibited a lower proportion of self-related elements (M = 0.14, SD = 0.02) compared to young adults (M = 0.16 SD = 0.02), *t* (101) = 2.81, *p* = .006, *d* = 0.55. However, there was no significant difference between young adults (M = 0.10, SD = 0.03) and older adults (M = 0.10, SD = 0.02) in proportion of other-related elements, *t* (101) = 1.18, *p* = .24, *d* = 0.23 (Fig. 4C and D). Results for the ratio measure for other to self-related elements are consistent with the proportion findings and can be found in Supplementary Figure S2.

*The Mind’s Eye Mind’s Mind Scoring Protocol Demonstrates Weak but Significant Relationships with the Autobiographical Interview*.

Correlations between proportion scores for the mind’s eye and internal details (i.e., internal details: total details) were positively correlated, *r* (101) = 0.26, *p* = .007, as well as proportion scores for mind’s mind and external details (i.e., external details: total details), *r* (101) = 0.22, *p* = .03. The cross correlations were also significant such that the mind’s eye and external details were negatively correlated, *r* (101) = − 0.26, *p* = .006 and mind’s mind and internal details were also negatively correlated, *r* (101) = − 0.22, *p* = .03.

### Study 1 discussion

Consistent with our hypotheses, Study 1 results revealed that cognitively healthy older adults, compared to young adults, show a relatively greater use of the mind’s mind, in particular content-general elements, and a decreased use of the mind’s eye, when retrieving autobiographical event memories. In addition, results show that the narrated autobiographical memories of older adults, compared to young adults, are related less to the self. In addition, we show that the Autobiographical Interview scoring protocol and the Mind’s Eye Mind’s Mind scoring protocol are significantly, albeit weakly, correlated. We next turn to Study 2, in which we examined (1) whether the mind’s eye and mind’s mind are similarly called on during past and future forms of imaginative event thinking among older adults, and (2) whether there is a bias to use the mind’s mind and to focus on others with advanced older age.

### Study 2

### Methods

### Participants

Study 2 was a secondary analysis of an experiment involving remembering past events and thinking about hypothetical future events (for details see: Acevedo-Molina et al., 2023). Eighty-five individuals between the ages of 60–80 years old participated in this study. Two participants were discontinued because they did not understand study task procedures. One other participant did not return for the second session. Thus, 82 total remaining participants were analyzed and reviewed in this study. Demographic and neuropsychological data for these participants are illustrated in Table 1. This dataset was enriched with carriers of the ε4 allele of the apolipoprotein E gene (APOE)^56^. Participants were assessed as clinically normal based on a comprehensive battery of neuropsychological tests^51^, using the same criteria to determine eligibility as Study 1. All participants were screened for self-reported neurological and history of psychiatric conditions that could have cognitive effects. Participants had to score below the cutoff score of 16 on the Center for Epidemiological Studies Depression Scale indicative of risk for clinical depression^57^ and report being independent in activities of daily living. Informed consent was obtained from all participants. All study procedures were performed in accordance with relevant guidelines and regulations and approved by the University of Arizona Institutional Review Board.

###  Procedures

### Past and future thinking task

The past and future thinking task, adopted from Addis et al., 2010^49^, was spread over seven days with two different sessions. The purpose of the first session was to gather memories that could serve as past or future thinking trials. Participants were asked to generate 16 memories from the past five years and were told that each memory must contain what they considered a “very vivid and detailed” person, object, and place and that these details needed to be unique for each memory. To encourage retrieval, participants were cued with 16 pictures (selected via Google image searches), which generally corresponded to the meaning of 16 words selected from the Clark and Paivio norms^58^ that were scored high on frequency (mean = 1.84), imageability (mean = 5.91), and concreteness (mean = 6.71). Participants were informed that they need not rely on the pictures to cue retrieval. The first trial was used for practice and discarded, leaving 15 memory generation trials to be used for Session 2.

Between session one and two, an experimenter randomly selected five memories to serve as memory (past thinking) trials. These person-object-place detail triplets were preserved. The future thinking trials were created by recombining the people, objects, and places from the remaining ten memories. The experimenter ensured that each recombined triplet was entirely new. Five of these recombined triplets were randomly selected as future thinking trials, two were selected for practice, and three were available in case a selected triplet was reported as improbable.

In the second session, participants were told that they would need to recall some memories (past thinking task) that they generated in session one, in addition to thinking of several new events that could happen in the next five years (future thinking task). Participants were shown a triplet of a person, place, and object and were asked to either recall the previously generated past event from session one or think of a future event happening in the next five years with a recombined triplet from the memory task. The order of the past and future thinking trials was quasi-randomized so that no more than two trials of either task were presented sequentially. Participants were instructed to focus on the unique features of the event and to include the three unique details from the triplet. If the participant was presented with an improbable event on a future thinking trial (e.g., a person who had passed away), they informed the experimenter, who would then select a new triplet. Each participant was given four minutes per trial. The participant’s responses were audio recorded then subsequently transcribed. Raters did not score past the four-minute mark.

### Scoring procedure & reliability

Participants completed ten narratives (five for past thinking and five for future thinking). Eleven participants completed only four narratives for memory and four for future thinking due to time constraints. The same metrics as in Study 1 were extracted from both the past and future narratives using the same procedure and protocol. As with Study 1, Study 2 also had two trained raters (authors MH and NC) who scored the transcripts and were unaware of participant age. However, in Study 2, rater MH scored all the transcripts and rater NC scored 24% of the transcripts to calculate reliability. The analyzed data was derived from rater MH’s scoring. The reliability was excellent for mind’s mind-past (ICC = 0.97), mind’s eye-past (ICC = 0.98), mind’s eye-future (ICC = 0.97), and mind’s mind-future (ICC = 0.96). Reliability of self- and other-related elements was excellent for self-past (ICC = 0.92) and good for other-past (ICC = 0.87), self-future (ICC = 0.82), and other-future (ICC = 0.84). The reliability of non-imaginative thoughts was moderate for the past thinking task (ICC = 0.60) and for the future thinking task (ICC = 0.66).

### Statistical analyses

Normality of the data was dealt with in the same way as in Study 1. Effect sizes are reported as Cohen’s d for t-tests and generalized eta squared for ANOVA results. For ANOVAs, we used a Bonferroni correction on planned post hoc comparisons. Analyses were performed in R^54^. Figures were created in R using ggplot2^55^.

Consistent with Study 1, we examined both proportion and ratio measures and found that the ratio measure had a strong and positive correlation with the proportion results for both future thinking (mind’s mind *r* (80) = 0.99 and mind’s eye *r* (80) = 0.97, *p* values < 0.001) and past thinking (mind’s mind *r* (80) = 0.99 and mind’s eye, *r* (80) = 0.97, *p* values < 0.001). For conciseness, we provide the ratio results in the Supplementary Information.

To examine whether the categories (i.e., mind’s eye and mind’s mind) were being used to a similar extent across both tasks (i.e., past and future thinking tasks), we conducted a Pearson correlation using the proportion of mind’s mind and mind’s eye elements in each task. Then, we conducted a two-way repeated measures ANOVA using proportion values with factors for category of imaginative thought (mind’s eye and mind’s mind) and task type (past and future thinking), as this could inform whether the relative weighting of the mind’s eye and mind’s mind differs between past and future thinking.

To examine whether advanced age is associated with a shift toward the mind’s mind in both past and future thinking, we conducted a Spearman correlation (given that age was not normally distributed) using the proportion measure and age as a continuous variable. To assess if older age is associated with a shift toward thoughts related to others, we conducted a Spearman correlation between the proportion of other-related elements and age for both the past and future thinking tasks. To analyze if there were differences in production of self- versus other-related information in past and future thinking, we conducted a two-way repeated measures ANOVA using proportion values with factors for self-other orientation (self vs. other) and task type (past vs. future). We also conducted these analyses using the ratio measure. Given that the correlation between the proportion and ratio measures were significantly correlated for both past thinking (self *r* (80) = 0.60 and other *r* (80) = 0.73, *p* values < 0.001) and future thinking (self *r* (80) = 0.78 and other *r* (80) = 0.60, *p* values < 0.001), we provide the ratio results in the Supplementary Information.

### Study 2 results

### Older adults show relatively stable use of mind’s mind with advanced older age

**Fig. 5:**
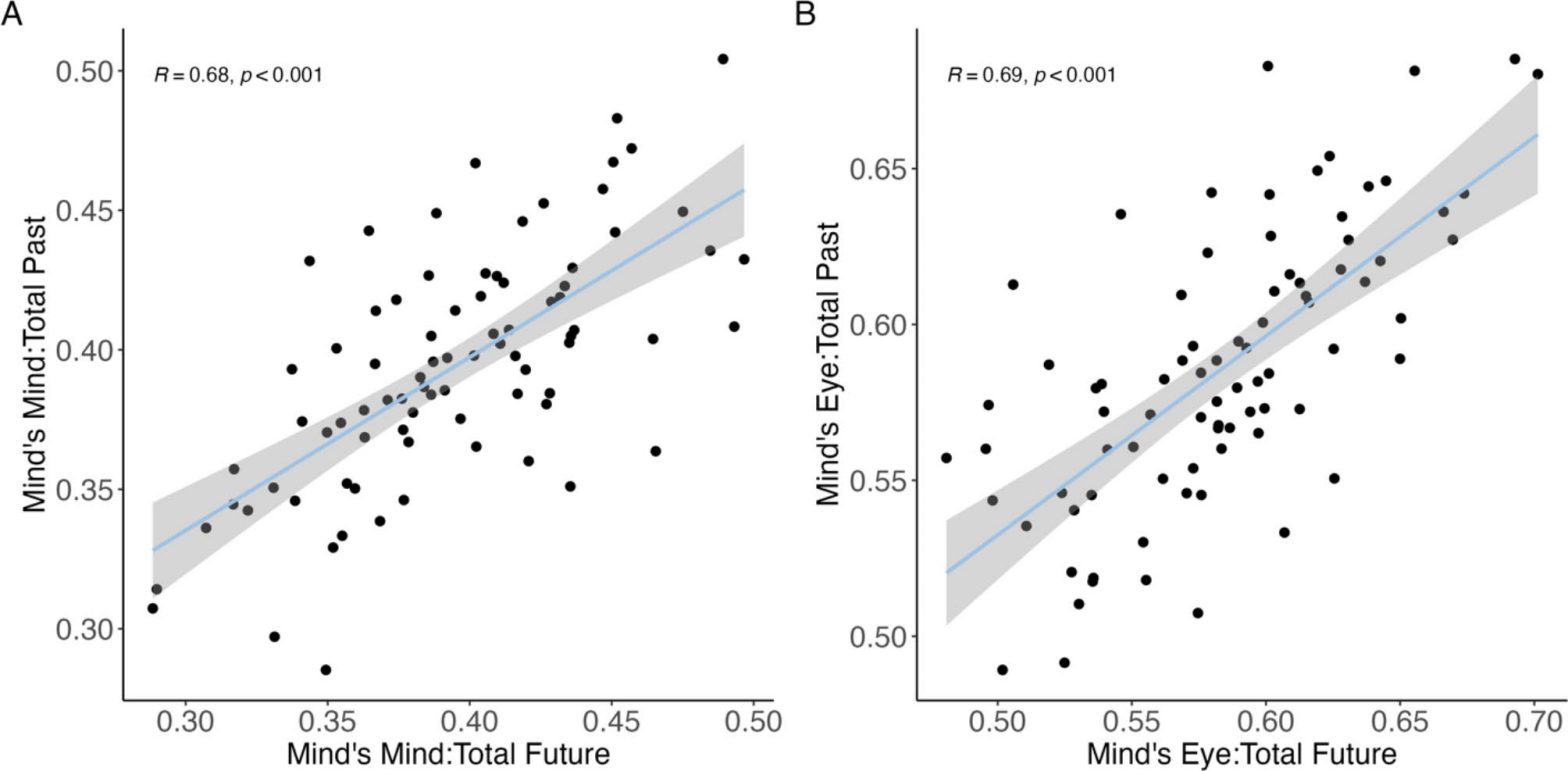
Pearson correlation between proportion of mind’s mind and mind’s eye elements for past and future thinking. The shaded region represents the 95% confidence interval of the regression line.

Descriptive statistics for the proportion of mind’s eye and mind’s mind elements and ratio of mind’s mind to mind’s eye elements are reported in Supplementary Tables S2 and S3. Regarding the relationship between the past and future thinking tasks, we found significant, moderate-to-large correlations for the proportion of mind’s mind elements, *r* (80) = 0.68, *p* < .001 and mind’s eye elements, *r* (80) = 0.69, *p* < .001 between both tasks (see Fig. 5). This suggests that participants who tend to use more mind’s mind elements (as well as mind’s eye elements) to describe past experiences, also tend to use more mind’s mind elements (as well as mind’s eye elements) to describe hypothetical future events. The ratio measure shows consistent findings and can be seen in Supplementary Figure S3.

The two-way repeated measures ANOVA did not show a main effect of task type, *F* (1,81) = 1.06, *p* = .31, η^2^_*G*_ = 0.00009, such that the total proportion of imaginative thought elements did not significantly vary between past and future thinking. Similar to Study 1, we observed a significant main effect of category of imaginative thought, *F* (1,81) = 434.12, *p* < .001, η^2^_*G*_ = 0.81, such that there was a greater proportion of mind’s eye elements (M = 0.58, SD = 0.05) than mind’s mind elements (M = 0.39, SD = 0.04). Critically, we did not observe a significant interaction between task type and category of imaginative thought, *F* (1,81) = 0.13, *p* = .72, η^2^_*G*_ = 0.00024, indicating that the relative differences in proportion of mind’s eye and mind’s mind elements did not vary across the past and future thinking tasks. A follow up Repeated Measures Bayesian ANOVA revealed substantial evidence for a lack of an interaction between mind’s mind and mind’s eye in past and future thinking, BF_10_ = 0.17. The ratio measure showed consistent results regarding the use of the mind’s mind relative to the mind’s eye in past and future thinking (see Supplementary Figure S3).

Correlations between age and proportion of mind’s mind and mind’s eye elements are shown in Supplementary Figure S4. Regarding the correlation between proportion of mind’s mind elements and advancing age among older adults, we did not find a significant relationship for either the past thinking task, *ρ* (80) = 0.009, *p* = .94 or the future thinking task, *ρ* (80) = 0.13, *p* = .23. Similarly, we did not find a significant relationship between the proportion of mind’s eye elements and advancing age for the past thinking task, *ρ* (80) = − 0.1, *p* = .39 or the future thinking task, *ρ* (80) = − 0.15, *p* = .18. The ratio measure was consistent with the proportion results and can be found in Supplementary Figure S5.

*Although older adults show a tendency to reference others more than the self in past and future thinking*,* this tendency was not associated with advanced age*.

Descriptive statistics for the ratio of mind’s mind to mind’s eye elements and proportion of self- and other-related elements can be found in Supplementary Tables S3 and S4. The relationships between the proportion of self- and other-related elements and advancing age among older adults can be seen in Supplementary Figure S6. Advanced age was not significantly related to the proportion of other-related elements for the past thinking task, *ρ* (80) = − 0.04, *p* = .75, or the future thinking task, *ρ* (80) = − 0.08, *p* = .46. In addition, advanced age was not significantly related to the proportion of self-related elements in either the past thinking task, *ρ* (80) = − 0.04, *p* = .73 or future thinking task, *ρ* (80) = 0.007, *p* = .95. Results for the ratio measure show consistent findings and can be seen in Supplementary Figure S7.

A two (self vs. other) by two (past vs. future) repeated-measures ANOVA revealed a significant main effect of self-other orientation, *F* (1,81) = 218.91, *p* < .001, η^2^_*G*_ = 0.53 but not task type, *F* (1,81) = 3.56, *p* = .063, η^2^_*G*_ = 0.005. Specifically, we observed a greater proportion of other-related elements (M = 0.13, SD = 0.03) than self-related elements (M = 0.08, SD = 0.02). However, the proportion of referenced elements did not significantly vary between past and future thinking. In addition, we observed a significant interaction between task type and self-other orientation, *F* (1,81) = 19.76, *p* < .001, η^2^_*G*_ = 0.033. Post-hoc t-tests revealed a greater proportion of other related elements in the future thinking task (M = 0.14, SD = 0.03) compared to the past thinking task (M = 0.12, SD = 0.03), *t* (81) = 3.87, *p* < .001, *d* = 0.43, and a lower proportion of self-related elements in the future thinking task (M = 0.07, SD = 0.02) compared to the past thinking task (M = 0.08, SD = 0.02), *t* (81) = 2.48, *p* = .015, *d* = -0.27. The ratio results, which are consistent with the proportion findings related to self-other orientation, can be found in Supplementary Figure S7.

### Study 2 discussion

Three important findings emerged from Study 2. First, we showed that the mind’s eye and mind’s mind, as measured by our novel scoring protocol, were used consistently across two types of imaginative thinking among older adults. This was demonstrated by moderate-to-strong correlations in the use of mind’s eye and mind’s mind process when remembering the past and thinking about the future, and in the relative use of the mind’s eye and mind’s mind across past and future thinking. Second, we did not observe a change in the use of either the mind’s eye or mind’s mind with advancing age among older adults. Lastly, and counter to our hypotheses, advanced older age was not associated with a shift in the proportion of other-related thoughts or the ratio of other- to self-related thoughts.

## General discussion

The present study sought to close the gap in knowledge regarding how the mind’s eye and mind’s mind components of imagination are expressed in autobiographical past and future thinking in young and older adults. To investigate a proposal informed by the broad literature on imagination and the default network^3^, we developed and applied a novel scoring protocol to measure the relative use of two processes, namely the mind’s eye and the mind’s mind, in two forms of imagination, past and future thinking. We applied this scoring protocol to a study examining imaginative thinking in young and older adults, and a separate study consisting of only older adults. There were four main findings. First, we demonstrate that the use of mind’s eye and mind’s mind is consistent with the framework we proposed, including reliability in objective scoring (Study 1 and Study 2) and consistency in how these two cognitive processes are called upon while engaged in various forms of imaginative thought (i.e., past and future thinking, Study 2). Second, we found a significant age-related shift in the balance of mind’s eye and mind’s mind. Although young and older adults both used mind’s eye more than mind’s mind in Study 1 (possibly influenced by the nature of the task), this difference was significantly weaker in healthy older adults, which appeared to be driven by greater use of content general elements. Third, the relative use of the mind’s eye and mind’s mind was stable in relation to advanced older age in Study 2. Lastly, although both young and older adults had a greater proportion of self-related thoughts compared to other-related thoughts in Study 1, this difference was significantly weaker in healthy older adults, suggesting less of a self-focused bias in older adults. Interestingly, in Study 2, the older adult cohort showed a clear bias toward other-related thinking, but the relative referencing of self and others was stable with advanced older age. Below we discuss the implications of these findings and directions for future research.

To address our first study goal of developing a novel scoring protocol for mind’s eye and mind’s mind, we examined reliability not only in how raters apply the scoring protocol, but also in how participants draw on content to engage related forms of imaginative thinking. Regarding experimenter reliability, both studies showed high interrater reliability across multiple raters (ICC’s ≥ 0.90) for mind’s eye and mind’s mind. Likewise, in Study 2, we found that older adults engaged the mind’s eye and mind’s mind in similar ways across two different forms of imagination (i.e., remembered events versus future event thinking), as shown by moderate to strong significant correlations between the proportion of mind’s eye and mind’s mind elements. Furthermore, consistent with the instructions to be specific in the event retrieval and future thinking tasks in both Study 1 and 2, a significantly greater proportion of thoughts were classified as mind’s eye than mind’s mind. This is noteworthy given that there were significant methodological differences across studies, including the timeframe of imagination (i.e., past or future) and the content or nature of what was to be imagined. In addition, the proportion of mind’s eye and mind’s mind did not vary based on the type of task (i.e., past versus future thinking) in Study 2. Taken together, these results support several key aspects of the Mind’s Eye Mind’s Mind framework and provide evidence for the stability and reliability of the scoring protocol.

In terms of the second goal to understand how mind’s eye and mind’s mind forms of imagination shift in older age, results reveal that although both groups in Study 1 showed a bias for mind’s eye thinking, this bias was statistically weaker for healthy older adults. Older adults, compared to young adults, used the mind’s mind relatively more regardless of whether we examined ratio or proportional measures. In the context of autobiographical event remembering, the content general element subtype of the mind’s mind was mainly responsible for the difference between young and older adults. Consistent with the literature on event-based forms of autobiographical memory, these findings suggest that older adults compared to young adults show a tendency to use more abstract, conceptual information^48,59,60^, and less specific contextual details^61,62^. This might be associated with age-related changes in the default network during autobiographical memory and episodic future thinking, which may affect the MTL subsystem more than the dmPFC subsystem in typical older age^33,37,63,64^.

In regard to shifts within older adults, Study 2 did not reveal changes in the relative use of mind’s mind and mind’s eye across older age in either the past thinking task (i.e., when they were asked to recall memories from the past five years) or the future thinking task (i.e., when they were asked to come up with events that could happen in the next five years). This finding might suggest that in typical older age, the use of mind’s mind and mind’s eye is relatively stable. This is consistent with work that has highlighted the relatively stable, and even enhanced, use of conceptual and gist information in normal aging^38,65,66^. This finding may also be related to work on the default network which has shown that the dmPFC subsystem is involved in more abstract conceptual processing^16^, which might be more strongly recruited in older adults in autobiographical past and future thinking^33^. However, more work is needed to assess default network subsystem changes in older age, and how this might contribute to a shift toward the mind’s mind. Notably, we used a recombination task (Addis et al. 2010) that may limit the role of abstract future thinking, as the task controls temporal distance and the primary content of the imagined event.

Another major finding from the present study was that both young and older adults differ in the extent to which they refer to themselves (self-related) and to other people (other-related). Although in Study 1 both young and older adults showed a greater proportion of self-related thoughts, older adults showed less of a self-focused bias than young adults. Within the older adult cohort in Study 2, older participants’ relative use of self and other reversed such that older adults showed a clear bias toward other-related thoughts compared to self-related thoughts, possibly because the task in Study 2 required participants to include another person in the memory or future event. However, in Study 2 advanced older age was not significantly related to the proportion of other-related thoughts or the ratio of other- to self-related thoughts, suggesting that thoughts related to the self and others may be stable in healthy older age. These findings add to the relatively little data on content of thoughts in older adults. In particular, our findings align with work in spontaneous thinking and experience sampling, which has shown that older adults demonstrate less self-referential thoughts than young adults^42,43^. In contrast, the current study aligns less with work in resting-state contexts, where there is no explicit task and participants are asked to retrospectively evaluate their thoughts, which has revealed no significant age differences between self-focused versus other-focused thoughts^47^. These findings can be couched in the broader literature on the socioemotional goals in older age, in particular, that older adults prioritize emotionally important relationships due to changing time horizons^40,46,67,68^. Nonetheless, findings from the current study and prior literature suggest there may be contexts in which young and older adults differ in how much they reference themselves and others. The current scoring protocol provides a novel and accessible way to objectively assess these thoughts in different forms of imaginative thinking in both young and older adults.

Although the present study provides key behavioral insights, it also raises new questions for future research. For instance, an important future direction of this work will involve examining whether the subsystems of the default network^3,4^ support the mind’s eye and mind’s mind as hypothesized. Default network subsystem involvement in different forms of thought might be particularly important in older populations, as the default network is targeted in neurodegenerative disease^37^. Relatedly, the Mind’s Eye Mind’s Mind framework posits that these two forms of thought are distinct but can interact when the subsystems of the default network are functional. This might mean that certain features of the mind’s mind may prompt certain features of the mind’s eye, and vice versa. Our correlation analyses, and the corresponding preliminary exploratory factor analysis, may hint at element types that tend to prompt interaction, but future work could more directly explore this hypothesis using temporally dynamic approaches, as well as examine if the two subsystems dissociate in clinical populations for whom one type of imaginative thought may be disrupted (e.g. hippocampal amnesia may disrupt the mind’s eye which might mean a stronger mind’s mind). Although our scoring protocol aims to capture the representational form of imaginative thinking as either more verbal or visual, one potential limitation is that the scoring protocol makes an inference based on language. If a person expresses that they love their dog, our scoring protocol would infer that the reference to the dog is a concrete concept which may or may not be associated with visual imagery. A future study may therefore combine behavioral and functional neuroimaging methods and/or corroborate the findings with self-reported phenomenology to refine the Mind’s Eye Mind’s Mind framework. The current study also focuses primarily on the self/other distinction given prior work showing age related changes in this aspect of thought. However, other features of thought such as emotional characteristics and focus on personal goals may also provide insight for understanding age related changes in imaginative thinking. Future work should explore these other features of thought in different types of imaginative thinking.

Another important future direction for this work involves examining how the mind’s eye and mind’s mind function in unprompted or other forms of imaginative thinking. For example, periods of unprompted imaginative thought, where participants vocalize what is on their minds in the moment, can provide information not granted by tasks with specific instructions^43,47,69^. This includes how the content of thoughts can be captured dynamically^70^, which may reveal different characteristics related to trait brooding^71^ and creativity^72^, and can further elucidate age differences in thought content^73^. Indeed, similarities between the cognitive and neural processes that support different forms of thinking have led to a shift in perspective on what constitutes imaginative thought^5,7,74,75^. The novel scoring protocol presented here provides a valuable tool with which to characterize these thoughts and capture age-related changes that occur in different forms of imaginative thinking.

To further evaluate the psychometric properties of the Mind’s Eye Mind’s Mind scoring protocol, we generated a correlation matrix and conducted an exploratory factor analysis on total count data (see Supplementary Information), similar to Lockrow et al.^76^. Notably, the Mind’s Eye Mind’s Mind framework does not stipulate that all subtypes of one form of imagination must be positively correlated or negatively correlated with the other form of imaginative thought. In fact, it is likely that there are cross category correlations, given that the mind’s eye and mind’s mind are presumed to be highly interactive in healthy cognition, especially during the temporal unfolding of autobiographical narratives. Future research is needed to determine whether the pattern and magnitude of correlations between element subtypes is consistent across forms of imagination and whether the element subtypes load on to the default network subsystems in line with the main tenant of the theory. Until these questions are addressed, we recommend that users of the Mind’s Eye Mind’s Mind scoring protocol analyze element subtypes, in addition to aggregated mind’s eye and mind’s mind scores.

Several scoring protocols have emerged in recent years to understand the content of event memories^77,78^. Consistent with this work, we sought to understand how the Mind’s Eye Mind’s Mind scoring protocol relates to the event, detail-based scoring of the Autobiographical Interview. Our analyses showed that there are significant, albeit small, correlations between the proportion of elements defined as mind’s eye or mind’s mind and the proportion of internal and external details, as defined by the Autobiographical Interview scoring protocol. The relationships are consistent with the notion that the two scoring protocols treat certain elements or details similarly (e.g., internal details are specific to an event, with specificity being a quality of the mind’s eye). However, the fact that correlations were small in magnitude suggests that the two protocols have distinct features. One potential advantage of the Mind’s Eye Mind’s Mind scoring protocol is that it may not only measure event memories and future thoughts, but also other forms of imaginative thinking.

In conclusion, by applying a novel scoring protocol, we show that imaginative thinking can be reliably characterized as a function of the mind’s eye and the mind’s mind. This work adds to the growing body of research that highlights the interdependent nature of imaginative thought^5,7,28,74^. Here, we have shown that the mind’s mind and mind’s eye are instantiated differently between young and older adults and are applicable to different kinds of thinking (e.g., past and future). The scoring protocol introduced here is meant to capture different forms of imaginative thinking, including mind wandering and mentalizing. This novel model, if replicated and expanded, may provide an understanding of imaginative thinking that can perhaps guide clinical investigations into the different neurological and psychological disorders that might impact different aspects of imaginative thought.

## Electronic supplementary material

Below is the link to the electronic supplementary material.

Supplementary Material 1

## Data Availability

The datasets analyzed in this study are available at OSF: https://osf.io/356kh/.
