# Supplementary material for: A novel scoring protocol reveals age-related differences in abstract compared to concrete thinking in cued autobiographical remembering
Source: Sci Rep. 2024 Dec 27;14:30642. doi: 10.1038/s41598-024-82493-6 (PMC11680765; doi:10.1038/s41598-024-82493-6)
Supplement: Supplementary file 1 — Supplementary Material 1 [file 41598_2024_82493_MOESM1_ESM.docx]

**Supplementary Information**

A novel scoring protocol reveals age-related differences in abstract compared to concrete thinking in cued autobiographical remembering

Mariam Hovhannisyan^1*^, Quentin Raffaelli^1^, Nadine Chau^1^, Jessica R. Andrews-Hanna^1,2,3*^, Matthew D. Grilli^1,3,4*^

**Author Affiliations**

^1^Department of Psychology, University of Arizona, 1503 E University Blvd., Tucson, AZ, 85721, USA

^2^Cognitive Science, University of Arizona, Tucson, AZ, USA

^3^Evelyn F. McKnight Brain Institute, University of Arizona, Tucson, AZ, USA

^4^Department of Neurology, University of Arizona, Tucson, AZ, USA

**Supplementary Table S1:** Factor loadings for exploratory factor analysis in Study 1

| Element Type | Factor Loading | |
| --- | --- | --- |
|  | 1 | 2 |
| Concrete Concepts | **0.57** | **0.74** |
| Sensory | 0.08 | **0.67** |
| Visual | 0.20 | **0.63** |
| Location | **0.50** | **0.50** |
| Content Specific | **0.76** | 0.49 |
| Time | **0.74** | 0.24 |
| Content General | **0.64** | **0.61** |
| Inference Reasoning | 0.44 | **0.53** |
| Reflection Appraisal | **0.56** | **0.52** |
| Abstract Concept | **0.80** | 0.41 |
| Facts | **0.52** | 0.06 |

**Caption:** Factor loadings from exploratory factor analysis with varimax rotation. Factor columns are sorted by mind’s eye elements first then mind’s mind elements. Loadings greater than .5 are bolded.

**Supplementary Table S2:** Mean proportion of mind’s eye, mind’s mind, and non-imaginative elements for Study 1 and 2.

| Proportion of elements | | Mind’s Mind | Mind’s Eye | Non-imaginative/Task Related Inferences |
| --- | --- | --- | --- | --- |
| Study 1 | Older | 0.41 (0.03) | 0.56 (0.03) | 0.03 (0.01) |
|  | Young | 0.39 (0.03) | 0.58 (0.03) | 0.03 (0.02) |
| Study 2 | Past | 0.39 (0.04) | 0.59 (0.04) | 0.02 (0.02) |
|  | Future | 0.40 (0.05) | 0.58 (0.05) | 0.02 (0.02) |

**Caption**: Standard deviations are reported in parentheses.

**Supplementary Table S3:** Mean ratio of mind’s mind to mind’s eye elements and other- to self-related elements for both Study 1 and Study 2.

| Ratio of elements | | Mind’s Mind to Mind’s Eye | Other to Self – related |
| --- | --- | --- | --- |
| Study 1 | Older | 0.73 (0.09) | 0.74 (0.27) |
|  | Young | 0.68 (0.09) | 0.63 (0.24) |
| Study 2 | Past | 0.68 (0.12) | 1.61 (0.56) |
|  | Future | 0.69 (0.14) | 1.98 (0.93) |

**Caption:** Standard deviations reported in parenthesis.

**Supplementary Table S4:** Mean proportions of self-related and other-related elements accounting for all element types (i.e., mind’s eye, mind’s mind, and non-imaginative elements).

| Proportion of elements | | Self-related | Other-related |
| --- | --- | --- | --- |
| Study 1 | Older | 0.14 (0.02) | 0.10 (0.02) |
|  | Young | 0.16 (0.02) | 0.10 (0.03) |
| Study 2 | Past | 0.08 (0.02) | 0.12 (0.03) |
|  | Future | 0.08 (0.02) | 0.14 (0.03) |

**Caption:** Standard deviations are reported in parenthesis.

**Supplementary Figure S1:** Correlation matrix with count data in Study 1

**
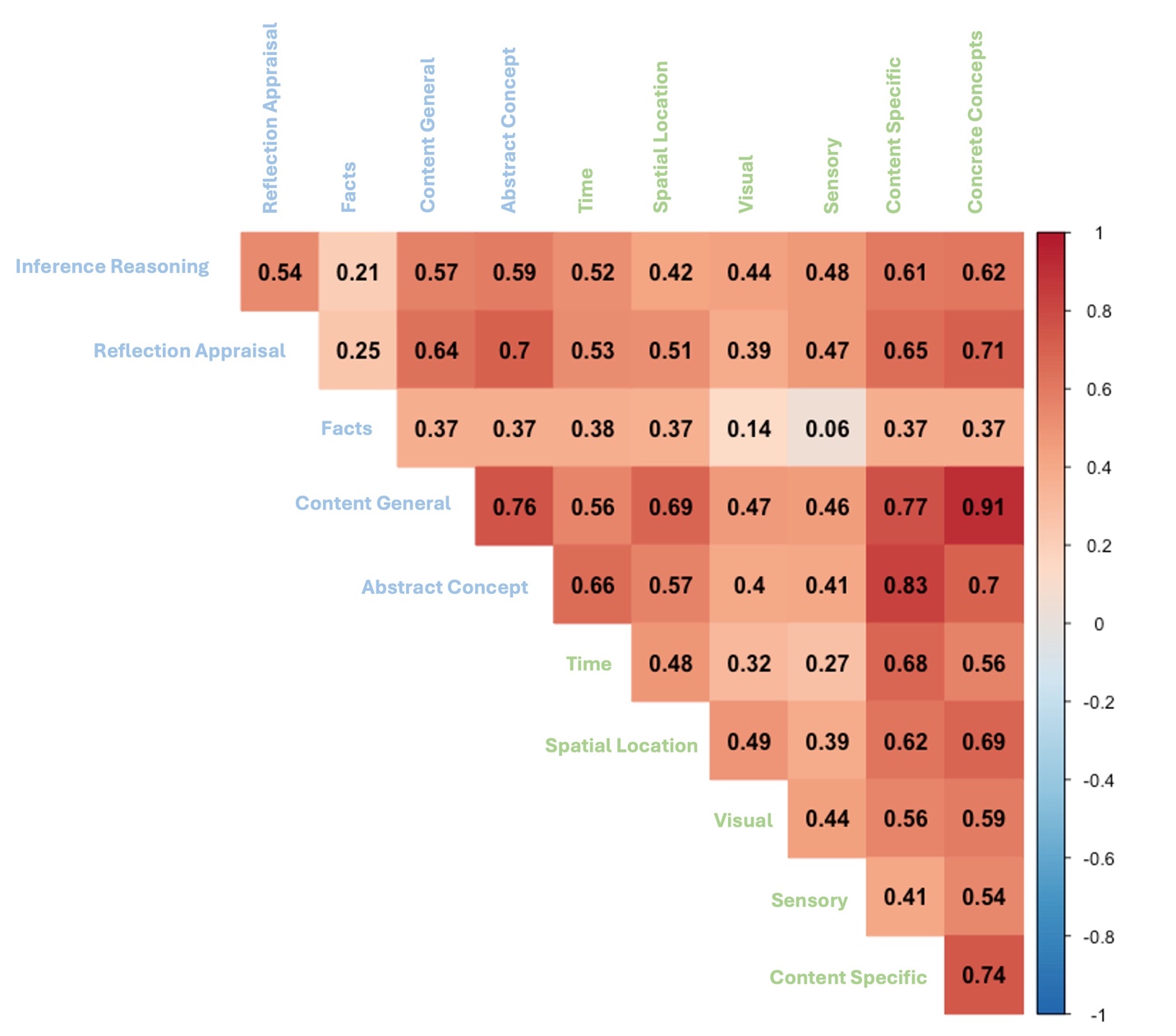
**

**Caption:** Spearman correlation between element subtypes of mind’s eye and mind’s mind variables. The matrix contains ρ values, which are color-coded by strength. All correlations are significant (p < .05, uncorrected) except for the correlation between Facts and Visual and Facts and Sensory.

**Supplementary Figure S2:** Age group differences for ratio of mind’s mind to mind’s eye elements and other- to self-related elements for Study 1.

**
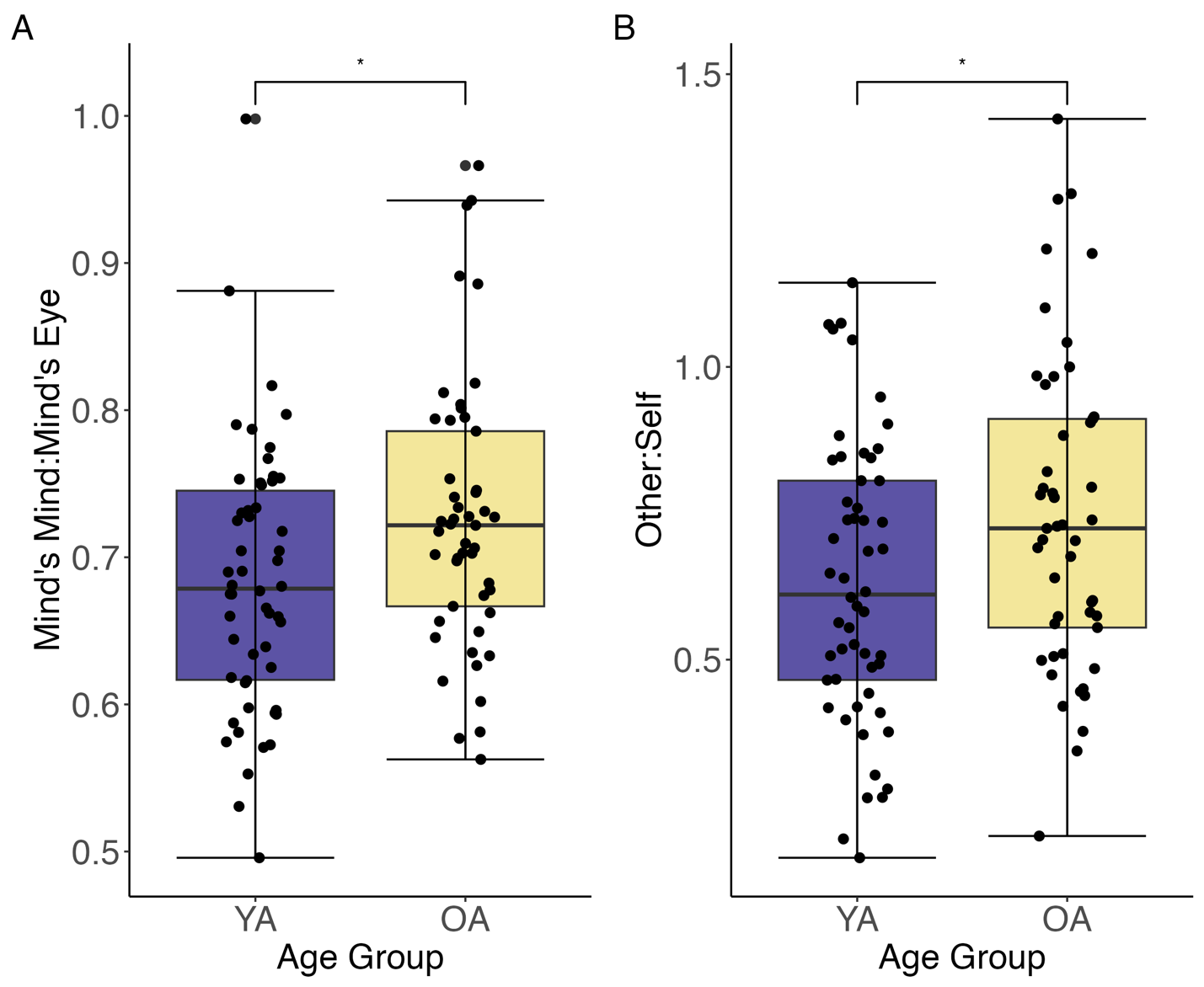
**

**Caption**: A) Ratio of mind’s mind to mind’s eye elements shows significantly greater ratio of mind’s mind compared to mind’s eye in older adults (M=0.73, SD=0.09) relative to young adults (M=0.68, SD=0.09), *t* (101) = 2.40 *p* =.02, *d* = 0.47. B) Ratio of other to self-related elements reveals a significant age group difference favoring older adults (M=0.74, SD=0.27) compared to young adults (M=0.63, SD=0.24), *t* (101) = 2.18, *p* = .03, *d =* 0.43. Boxplots show median represented by a line within each box. Brackets capture data within the upper and lower quartiles.

**Supplementary Figure S3:** Ratio of mind’s mind to mind’s eye elements between past and future thinking in Study 2.


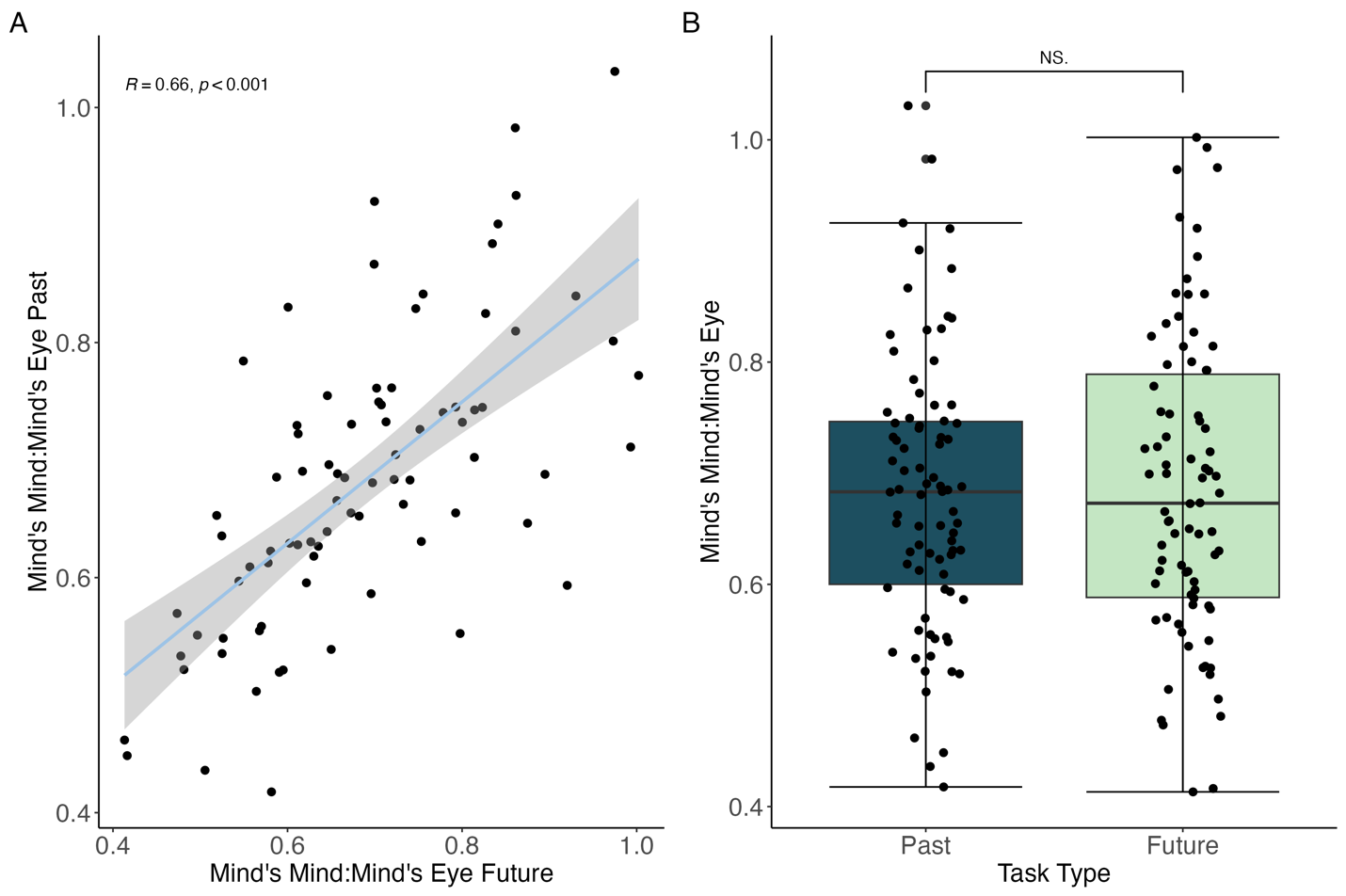


**Caption**: A) Relationship for ratio of mind’s mind to mind’s eye elements between past and future thinking**,** *r* (80) = .66, *p* < .001 and B) consistent use of mind’s mind to mind’s eye elements for both past and future thinking, *t* (81) = 0.45, *p* = 0.7, *d* = .05.

**Supplementary Figure S4**: Proportion of mind’s mind and mind’s eye elements across age in Study 2.
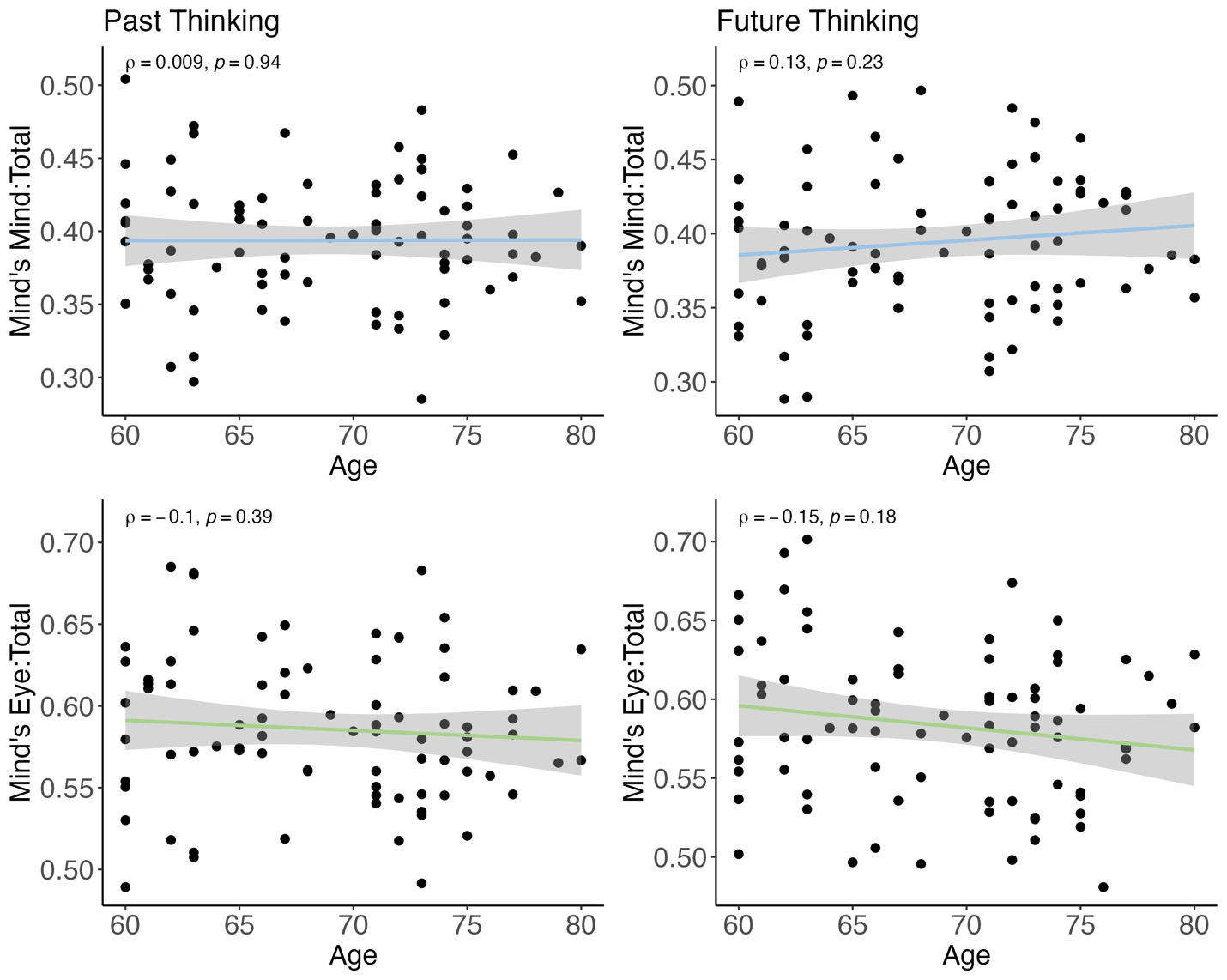
 **Caption**: Spearman correlation between age and proportion of mind’s mind elements (top row) and mind’s eye elements (bottom row) for past thinking (left) and future thinking (right) in Study 2. The shaded region represents the 95 percent confidence interval of the regression line.

**Supplementary Figure S5**: Relationship between age and ratio of mind’s mind to mind’s eye elements within older adults in Study 2.
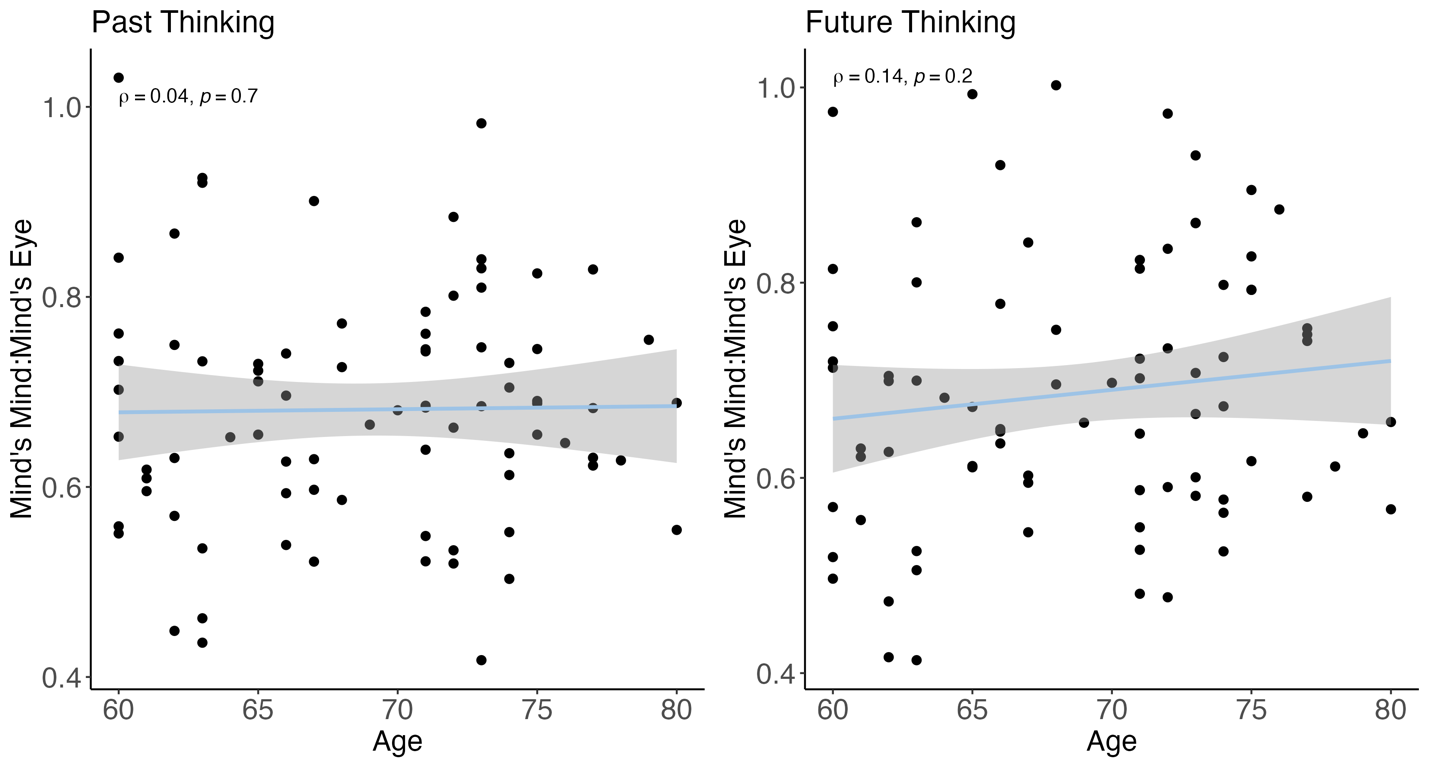
 **Caption:** Spearman correlation between age and ratio of mind’s mind to mind’s eye elements for past and future thinking. The shaded region represents the 95 percent confidence interval of the regression line.

**Supplementary Figure S6**: Proportion of self- and other-related elements across age in Study 2.
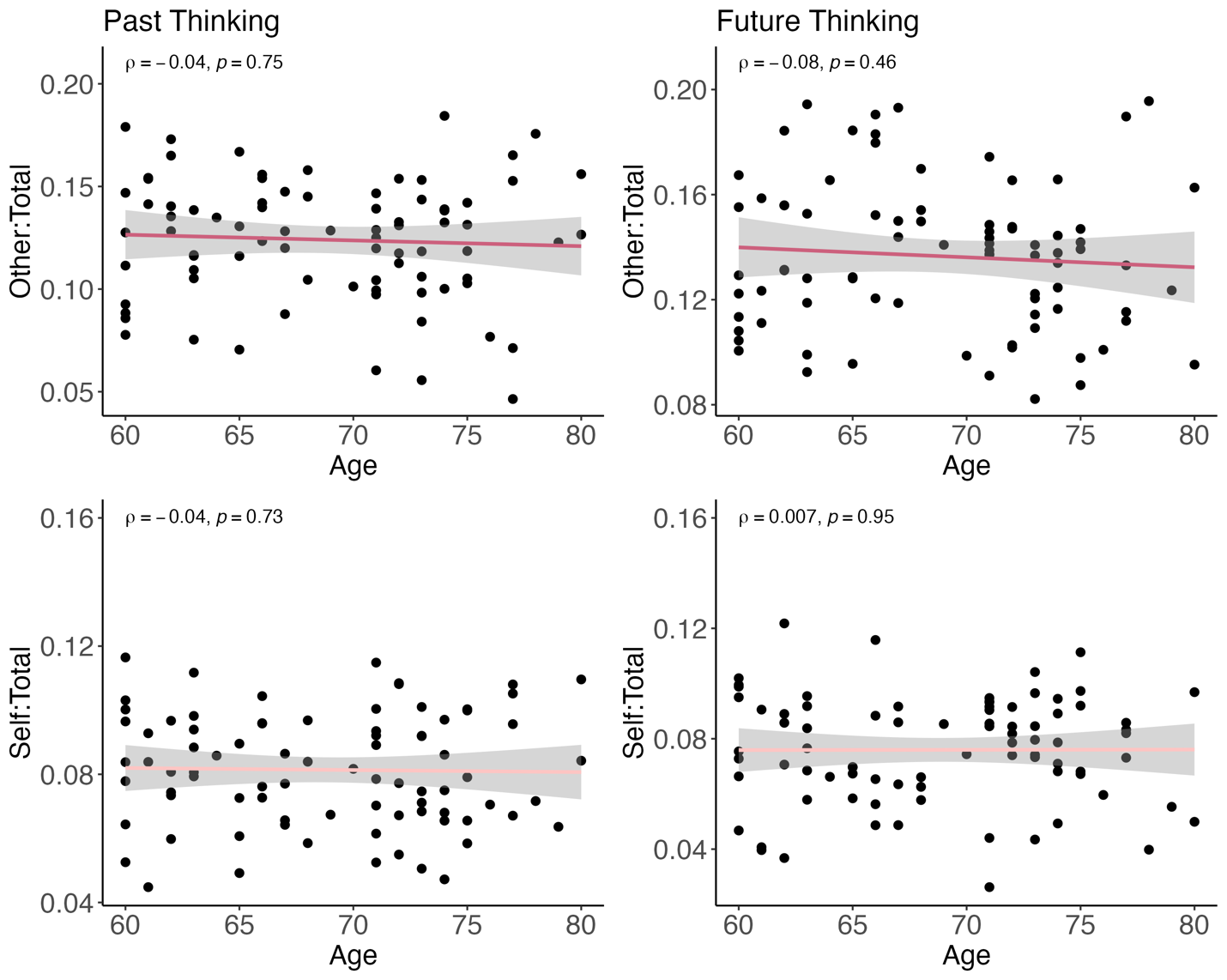
 **Caption**: Spearman correlation between age and proportion of other-related elements and self-related elements for past or future thinking. The shaded region represents the 95 percent confidence interval of the regression line.

**Supplementary Figure S7**: Spearman correlation of ratio of other to self-related elements in Study 2
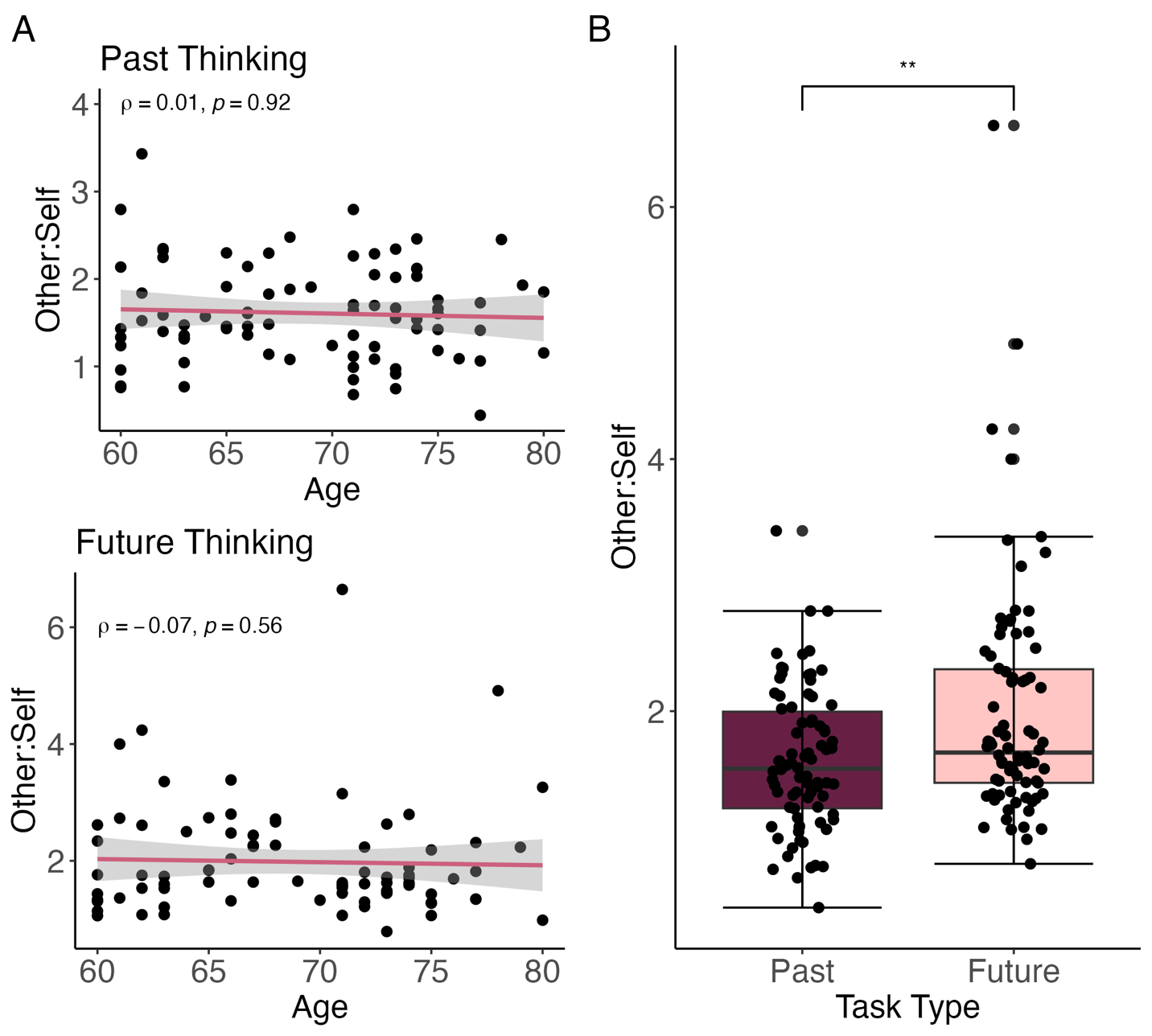
 **Caption:** A) Ratio of other- to self-related elements was not significantly correlated with advanced age for either the past thinking task or future thinking task. B) A significant difference was found between past and future thinking, *t* (81) = 4.47, *p* < .001, *d* = .49, such that future thinking (M=1.98, SD=0.93) showed a greater ratio of other- to self-related elements compared to past thinking (M=1.61, SD=0.56). Logarithmic transformation was applied to self and other data for the past and future thinking comparison due to non-normality. Figure shows untransformed data. The shaded region represents the 95 percent confidence interval of the regression line. Boxplots show median represented by a line within each box. Brackets capture data within the upper and lower quartiles.
